# ‘Something in the way she moves’: The functional significance of flexibility in the multiple roles of protein disulfide isomerase (PDI)

**DOI:** 10.1016/j.bbapap.2017.08.014

**Published:** 2017-11

**Authors:** Robert B. Freedman, Jasmine L. Desmond, Lee J. Byrne, Jack W. Heal, Mark J. Howard, Narinder Sanghera, Kelly L. Walker, A. Katrine Wallis, Stephen A. Wells, Richard A. Williamson, Rudolf A. Römer

**Affiliations:** a School of Life Sciences, Warwick University, Coventry CV4 7AL, UK; b Department of Physics, Warwick University, Coventry CV4 7AL, UK; c School of Biosciences, University of Kent, Canterbury CT2 7NJ, UK

**Keywords:** Protein dynamics, Protein disulfide isomerase, Oxidative protein folding, Endoplasmic reticulum

## Abstract

Protein disulfide isomerase (PDI) has diverse functions in the endoplasmic reticulum as catalyst of redox transfer, disulfide isomerization and oxidative protein folding, as molecular chaperone and in multi-subunit complexes. It interacts with an extraordinarily wide range of substrate and partner proteins, but there is only limited structural information on these interactions. Extensive evidence on the flexibility of PDI in solution is not matched by any detailed picture of the scope of its motion. A new rapid method for simulating the motion of large proteins provides detailed molecular trajectories for PDI demonstrating extensive changes in the relative orientation of its four domains, great variation in the distances between key sites and internal motion within the core ligand-binding domain. The review shows that these simulations are consistent with experimental evidence and provide insight into the functional capabilities conferred by the extensive flexible motion of PDI.

## 1 Introduction

An enzyme capable of catalysing the oxidative refolding of reduced ribonuclease *in vitro* was first described in the early 1960s; it was shown to catalyse the re-generation of ribonuclease activity, implying the formation of the unique set of ‘native’ disulfide bonds [1], [2]. The enzyme's systematic name – protein disulfide isomerase (PDI) – was first used in 1975 [3], and initially, evidence on its physiological role was limited [4]. But, by the mid-1980s, there were strong indications from its distribution, developmental profile and sub-cellular location that PDI plays a key role in forming disulfide bonds during the biosynthesis of secreted proteins and other disulfide-bonded proteins within the endoplasmic reticulum (ER) [5]. This important role was then confirmed by cross-linking studies in cells and sub-cellular fractions (see below), by reconstitution studies [6], and by the finding that, in yeast, the *PDI1* gene is essential [7]. The first sequence of a mammalian PDI (from rat) was determined in 1985 [8] and sequences of human and yeast PDI followed soon after [7], [9]. These sequences revealed that the enzyme is well conserved across eukaryotes and also highlighted some intriguing conserved features (see below).

PDI is abundant in mammalian secretory tissues such as liver, pancreas and placenta and a simple high-yielding purification procedure from such sources was developed in the early 1980s [10]. The cloning of cDNAs for mammalian and yeast PDI ensured that recombinant and mutant PDI could be expressed and purified in quantity [11]. So PDI became available as an object of study at a time when it was of considerable interest to the biotechnology industry in the context of its ability to facilitate the folding *in vivo* and refolding *in vitro* of very valuable pharmaceutical proteins. But despite this, and despite the fact that purified PDI is highly soluble and stable in solution, no high-resolution structure of PDI was published for many years. The first X-ray crystal structure of a full-length PDI (from the yeast *S. cerevisiae*) appeared in 2006 ([12]; PDB id: 2B5E), an alternative structure of yeast PDI was reported subsequently ([13]; PDB id: 3B0A) and structures of truncated human PDI were published in 2013 ([14]; PDB id: 4EKZ and id: 4EL1).

This long delay before structures were obtained from crystals of PDI strongly suggests that it is intrinsically flexible. This idea is also implied by two further key PDI properties, namely its multi-domain architecture and its remarkable diversity of biological roles and interactions. These properties are discussed in more detail in the next sections. The review then pulls together a large body of evidence indicating that PDI shows extensive flexibility in solution, introduces the results of simulating this flexibility by a new rapid method, and compares the predictions from these simulations to current experimental data. Finally, the review considers how the observed and predicted flexibility of PDI in solution could impact on its biological functions and discusses experimental methods that could now be applied to provide further detailed descriptions of PDI flexibility.

The review focusses on PDI from *Homo sapiens* (abbreviated hPDI) and from *Saccharomyces cerevisiae* (abbreviated yPDI). Where data on PDI from another species are cited, the species is specified. The general term ‘PDI’ is used where a statement is true across species. Both *H. sapiens* and *S. cerevisiae* express several homologs of PDI that are generally regarded as members of the ‘PDI family’ [15], [16] but since these have not been characterised structurally and functionally as thoroughly as the canonical PDI, we do not attempt to review evidence on their flexibility.

## 2 An outline of the overall architecture of PDI

**Fig. 1:**
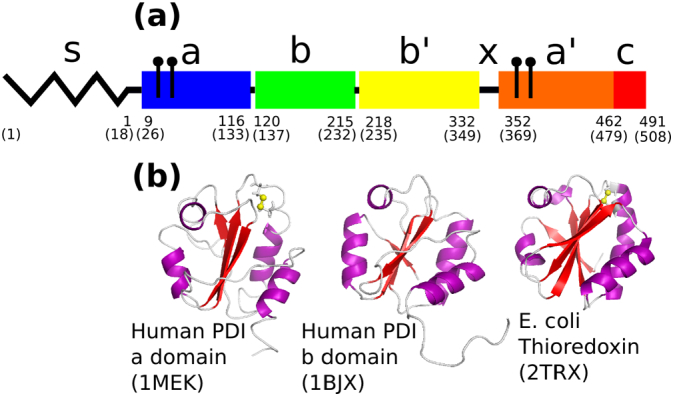
Domain architecture of PDI. a) Overall domain architecture of human PDI inferred from sequence homology, protease susceptibility and the properties of recombinant constructs representing putative domains [4], [5], [18], [26], [52]. The zig-zag (s) represents the signal sequence that is cleaved during biosynthesis and does not form part of the mature protein. Each coloured block (**a**, **b**, **b′**, **a′**) represents a domain of the mature protein. Note the original literature uses a residue numbering based on the sequence of mature PDI whereas some later literature uses a residue numbering based on the sequence of the inferred translation product (i.e. including the signal sequence). Residue numbers here and elsewhere in the review are for the mature sequence (above) and for the unprocessed translation product (below, in brackets). ¶ symbols represent cysteine residues of the active sites namely residues 36(53) and 39(56) in the **a** domain and 380(397) and 383(400) in the **a′** domain. b) Conservation of the thioredoxin-fold tertiary structure in the domains of PDI. The figure shows from left to right in the same orientation, human PDI **a** domain determined by NMR (PDB id: 1MEK), human PDI **b** domain determined by NMR (PDB id: 1BJX) and the archetype, *E. coli* thioredoxin, determined by X-ray crystallography (PDB id: 2TRX). The active site Cys residues in thioredoxin and PDI **a** domain are shown as ball-and-stick, with the disulfide highlighted in yellow. The PDI **b** domain does not contain this feature.

Repetitive features in the amino acid sequence of rat PDI [8] immediately suggested that it contained a number of domains (termed, in order, **a**-**b**-**b′**-**a′**), two of which probably resembled the simpler protein thioredoxin (termed **a** and **a′**) and two of which were of unknown structural type (**b** and **b′**). NMR studies on recombinant fragments by Creighton and collaborators then established the domain boundaries (Fig. 1a) and showed that all domains have the thioredoxin fold (trx-fold) (Fig. 1b), even those with no obvious sequence homology to thioredoxin [17], [19], [21]. The trx-fold is a tertiary structure element found in a wide range of proteins and comprises a central β-sheet of four or five strands flanked on either side by α-helices. The **a** domain of human PDI is highly homologous in structure to *E. coli* thioredoxin. It has an active-site –CGHC- sequence providing a dithiol/disulfide group at the location where trx-fold proteins frequently have their active site, in an exposed loop linking a β-strand to the following α-helix. Within this loop, the more N-terminal Cys residue is reactive and exposed to solvent while the more C-terminal Cys residue is buried. An acidic region at the C-terminus of PDI (**c** region) was recognised as lying beyond the thioredoxin-like domains, and it was also shown that there is a linker region, termed **x**, lying between the trx-folds of the **b′** and **a′** domains [18], [20]. Hence the overall architecture of mature PDI is **a**-**b**-**b′**-**x**-**a′**-**c**.

Distinct roles for the PDI domains were also described. The key enzymatic action of PDI is its capacity to catalyse thiol:disulfide oxidoreductions dependent on a dithiol/disulfide active site. So the identification of trx-fold domains containing the sequence –CGHC- immediately suggested roles for the **a** and **a′** domains in catalysis of thiol/disulfide chemistry [21]. Similarly, studies with peptide ligands showed that these ligands bound preferentially to the **b′** domain of PDI, and that they competed with the binding of unfolded proteins, but that all domains played a part in the binding of larger ligands [22]. Hence the overall architecture of PDI was established in outline before a high-resolution structure of the full-length protein was determined (Fig. 1). It was also clear that PDI operated by exploiting synergy between active sites located in the **a** and **a′** domains and a promiscuous ligand binding site whose core was located in the **b′** domain [23]. Later, high-field NMR work allowed the precise identification of the residues in the **b′** domain that interact directly with ligands [24].

## 3 PDI as catalyst of disulfide formation and isomerization in oxidative protein folding *in vitro*

PDI was initially identified as a ‘ribonuclease reactivating enzyme’ but its discoverers soon demonstrated its action in catalysing the oxidative re-folding and re-activation of several other small disulfide-bonded proteins. They proposed ‘… the hypothesis that the enzyme is a general and non-specific catalyst for disulfide interchange in proteins containing disulfide bonds’ [25]. This hypothesis has been amply confirmed; by 1984 more than twenty substrates of PDI oxidative refolding action *in vitro* were known [26]. This list included small single-domain proteins such as ribonuclease, lysozyme, and basic pancreatic trypsin inhibitor (BPTI) and also multi-domain proteins such as serum albumin and multi-chain proteins such as immunoglobulins and procollagen.

In these reactions PDI catalyses net formation of disulfide bonds and the source of oxidizing equivalents in these *in vitro* experiments can be a convenient small disulfide, such as oxidized glutathione (GSSG) or dissolved molecular oxygen, or hydrogen peroxide [27]. The reaction involves both disulfide formation and disulfide isomerization to correct non-native disulfides. The isomerization process can be studied in the absence of net oxidation by using randomly disulfide-bonded (or ‘scrambled’) proteins as substrates and again PDI shows wide specificity in this ‘isomerase’ activity. The action of PDI as catalyst of disulfide isomerization can also be observed in its ability to convert fully glutathionylated proteins into native proteins.

$$\text{Protein}\;{\left(\mathrm{SSG}\right)}_{\text{2n}}\to \text{Protein}\;{\left(\mathrm{SS}\right)}_{n}+n\;\text{GSSG}$$

These substrates are proteins which have been unfolded and reduced, with each free thiol then being reacted to generate a mixed disulfide with glutathione [28]. Finally, PDI can catalyse the net reduction of protein disulfide bonds. This reaction is most commonly observed when the protein substrate contains disulfides that are ‘meta-stable’ such as those of insulin, chymotrypsin, ricin and other proteins that are generated by proteolytic action on a precursor (proinsulin, chymotrypsinogen and proricin respectively). Whether the net effect on the protein substrate is oxidation, reduction or isomerization, all these reactions can be regarded as thiol:disulfide oxidoreductions or thiol-disulfide interchanges.

The proteins identified as substrates for PDI-catalysed thiol-disulfide interchange vary widely in size, charge, number of chains and domains, and in the fold of those domains. For example, basic pancreatic trypsin inhibitor (BPTI) is very small, comprising 58 amino acids linked by 3 disulfides into a compact domain roughly 3 nm × 2 nm × 2 nm, while human serum albumin (HSA) comprises 585 amino acids, with 17 disulfide bonds and is organised as 3 domains forming a solid equilateral triangle with triangular edges of 8 nm and a depth of 3 nm. For comparison, an immunoglobulin G (IgG) molecule comprising 4 chains and a total of 12 immunoglobulin-fold (Ig-fold) domains, contains 12 intra-domain disulfides, 4 or more inter-chain disulfides and has dimensions 10 nm × 10–15 nm × 2.5 nm. BPTI is basic (pI = 10.5) and contains very little helical backbone whereas HSA is acidic (pI = 5.5), 67% α-helical and all the disulfides link helices [29]. IgG molecules have a roughly neutral pI, are mainly β-sheet in secondary structure with the intradomain disulfides linking the two sheets within each Ig-fold domain. Hence there is extraordinary diversity among the proteins and protein environments in which PDI can catalyse thiol:disulfide interchange and formation of native disulfide bonds.

## 4 PDI as catalyst of oxidative protein folding *in cellulo*

Studies on cultured cells reveal a similar diversity and range of protein substrates for PDI action. The pioneering study was that of Roth & Pierce [30] who studied whether PDI was directly involved in the biosynthesis of an immunoglobulin M (IgM) in intact hybridoma cells. When these cells were depleted of glutathione (GSH) and analysed by immunoprecipitation with anti-PDI antibodies, direct linkage of PDI to the immunoglobulin chains via mixed disulfide bonds was detected, showing PDI acting enzymically on Ig chains. In cells with the normal reductive capacity of GSH, this direct linkage, was labile or transient. To detect such an interaction in these cells, a cleavable bifunctional cross-linker was used and immunoprecipitation of cross-linked cell material either with anti-IgM or anti-PDI antibodies demonstrated that both heavy and light chains had been cross-linked to PDI. The cross-linking of Ig chains to PDI was efficient and observed even at low levels of cross-linker, demonstrating that PDI was involved in direct interactions with Ig chains.

Subsequently PDI has been shown to be directly involved in the biosynthetic folding of a wide range of other proteins. Molinari & Helenius [31] showed that two viral membrane glycoproteins form transient mixed disulfides with PDI in virus-infected CHO cells, while Kellokumpu et al. [32] demonstrated the close association between PDI and its substrates procollagen I and III in skin fibroblasts. In thyroid cells whose main secretory product is the large dimeric protein thyroglobulin, transient folding intermediates of thyroglobulin are found disulfide-bonded to PDI [33]. More recently, using liver-derived hepatoma cell lines that secrete apolipoprotein B (an obligatory component of very low-density lipoproteins), Wang et al. [34] have shown that PDI directly interacts with the newly-synthesized ApoB via its redox-active sites, and assists in its oxidative folding.

A popular systematic approach to identifying PDI substrates *in cellulo* is ‘substrate-trapping’ using mutants of PDI in which active sites have been mutated to –CxxA- so that a permanent mixed-disulfide is formed between this mutant active-site and its protein substrate [35], [36], [37]. Using this approach in human hepatoma cells (HepG2), it was established that the major liver-cell-derived secretory proteins albumin, transferrin and α-fetoprotein are all substrates for PDI *in cellulo* [37]. This was confirmed by ‘knockdown’ experiments showing that the oxidative folding of these substrates was delayed *in cellulo* when cells are depleted of PDI [37]. But this may not apply to all oxidative folding substrates in all cells. In a human fibrosarcoma cell line (HT1080), few PDI substrates were detected by the ‘substrate-trapping’ approach although substrates were detected for other members of the human PDI family [35]. Similarly, work on mouse embryo fibroblasts showed that oxidative folding substrates that are membrane-tethered, interact preferentially *in cellulo* with a specific trans-membrane member of the PDI family [36].

Nevertheless, the specific examples quoted above imply that the oxidative folding of a very wide range of significant secreted and membrane proteins is directly facilitated by PDI. The range of PDI action in living cells is also shown by its widespread biotechnological exploitation in the expression of high-value recombinant proteins. Over-expression of PDI in yeast is a successful strategy for increasing production of valuable recombinant human proteins such as serum albumin and transferrin [38]. Surprisingly the approach is also useful in bacteria; directing recombinant human PDI expression to the cytoplasm or periplasm of *E. coli* allows the host bacterium to be more productive in generating folded recombinant human proteins (such as antibody fragments) in these heterologous environments [39].

The substrate range of PDI *in vivo* is clearly very wide but it is not limitless. Toxins from marine snails such as cone snails (Conus) comprise a vast diversity of disulfide-bonded proteins which include unique structural domains expressed only in conoidean venoms [40]. This rapid evolution of an unprecedented diversity of disulfide-bonded structural domains has been accompanied by the evolution of a large number of conotoxin-specific PDIs each expressed at high levels within the venom gland. This exceptional case, in which the evolution of new disulfide-bonded protein folds has been accompanied by the evolution of specific PDIs to fold them, emphasizes the fact that this is not the case in general — in most cells there is not a specific PDI to catalyse the folding of each type of protein fold since the ‘canonical’ PDI copes with most of them!

## 5 PDI as redox partner and molecular chaperone

**Fig. 2:**
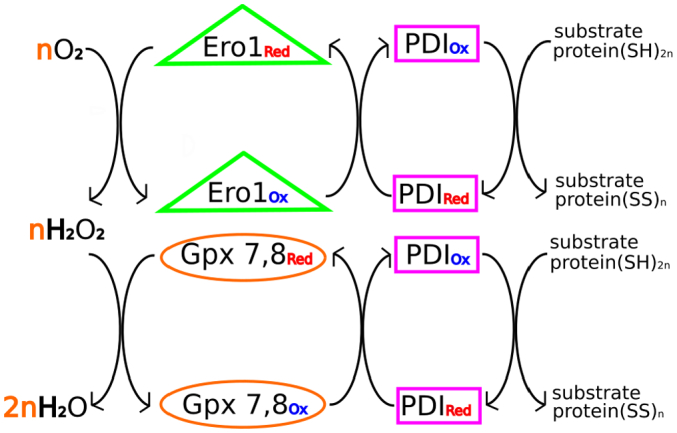
Role of PDI in electron transfer in the mammalian endoplasmic reticulum. Molecular O_2_ oxidizes reduced proteins in the ER via Ero and PDI, generating hydrogen peroxide (above); hydrogen peroxide can also oxidize reduced proteins via Gpx 7 and/or 8 and PDI (below). The Gpx7/8 pathway provides efficient use of oxidizing equivalents and detoxification of hydrogen peroxide. PDI interacts with both Ero and Gpx7/8 and, in both pathways, it acts on substrate proteins as the immediate oxidant and isomerase. In this representation, electrons flow from right to left, from a reduced protein to a terminal electron acceptor such as O_2_; alternatively, oxidizing equivalents can be considered to flow from left to right.

The previous section has highlighted PDI acting with very broad specificity on unfolded or partly folded protein substrates. In these actions, PDI functions as an oxidant and/or isomerase. *In vitro*, net oxidation is usually enabled by provision of oxidized glutathione (GSSG) to reoxidize reduced PDI. But the situation within the ER is different and more complex. To enable the continuous operation of PDI in oxidative protein folding, the re-oxidation of reduced PDI is performed primarily by specific flavoproteins that directly couple disulfide bond formation to the reduction of molecular oxygen. These ER-located oxidoreductins (Ero1p in yeast, Ero1α and Ero1β in mammals) transfer oxidizing equivalents from O_2_ to a flavin cofactor and then internally via a series of dithiol/disulfide groups to directly oxidize reduced active sites in PDI (for reviews see [41], [42]). The reaction also generates H_2_O_2_ and in higher organisms (but not in yeast) mechanisms exist to use this as an alternative oxidant to reoxidize reduced PDI. The mammalian ER contains several enzymes capable of catalysing this process [43], [44] but current evidence suggests that the key enzymes responsible for this recycling and detoxification are the ER-located glutathione peroxidases Gpx7 and Gpx8 [45], [46], [47]. Hence, in mammalian cells, PDI interacts directly with several oxidative redox partner proteins (see Fig. 2). In the case of Ero1α, there is some structural evidence on the nature of its redox transfer interaction with PDI [48], and also evidence that PDI is a key regulator of Ero1α, via redox interactions with ‘regulatory disulfides’ which constrain the enzymic activity of Ero1α [49].

In addition to these redox partners, PDI also participates in surprising cysteine-independent long-term interactions in the ER, as a component of specific functional complexes. Prolyl-4-hydroxylase (P4H), a key enzyme in the post-translational modification of collagens, exists in mammals as an α_2_β_2_ tetramer in which the α subunits are members of a wider Fe^2 +^- and α-keto-glutarate-dependent hydroxylase family, while the β subunits are PDI molecules [50]. Indeed human PDI was first cloned in a project to clone the P4H β subunit [9]. Similarly the microsomal triglyceride transfer protein (MTP) responsible for the insertion of triglycerides into lipoproteins is an αβ heterodimer of which the β subunit is PDI while the α subunit is a 97 kDa polypeptide related to the egg-yolk lipid-binding protein lipovitellin [51]. In both cases the functional sites are located in the α subunits, but PDI is not a passive additional component; it is responsible for the solubility and stability of the complex and for its initial stable assembly. In these complexes PDI appears to be acting as a molecular chaperone, assisting the folding of a partner protein; if so, it is a deviant chaperone which does not release its partner once folded but is required in long-term stoichiometric association.

The suggestion that PDI can act as a molecular chaperone is also supported by extensive work *in vitro*. CC Wang's group showed that PDI acts as a classic molecular chaperone suppressing aggregation and increasing folding yield during the refolding of several model proteins and suppressing aggregation during thermal denaturation [52]. The substrates used in these refolding studies were not disulfide-linked secretory proteins, so this chaperone activity is a demonstration of the capabilities of PDI rather than of its action *in vivo*, but there is good evidence that PDI does act as a chaperone *in vivo* during the folding and assembly of procollagen within the ER [53]. PDI is also found associated with misfolded protein aggregates in neurodegenerative disease states, suggesting that PDI may have a further chaperone-type role in modulating misfolding and aggregation of a variety of proteins [54].

Hence the number of proteins with which PDI interacts in living cells extends well beyond its wide range of oxidative folding substrates, to include redox partners, long-term functional partners and short-term chaperone substrates.

## 6 Structures of PDI obtained by X-ray crystallography

**Fig. 3:**
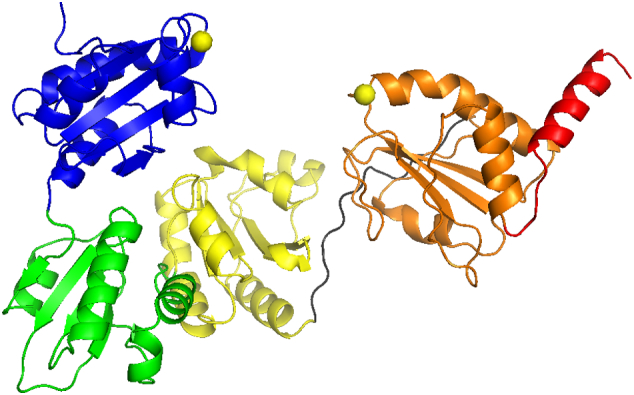
Tertiary structure of yeast PDI. Tertiary structure of full-length PDI from *S. cerevisiae* (2B5E) determined by X-ray crystallography [12]. Domains are coloured as in the outline domain architecture of Fig. 1, the exposed active site Cys residues in domains **a** and **a′** are indicated by yellow spheres and the **x**-linker region is indicated in dark grey.

The breakthrough crystal structure of yeast (*S. cerevisiae*) PDI determined by Schindelin, Lennarz and colleagues [12] provided the first high-resolution structure of a full-length PDI (PDB id: 2B5E) and the first clear picture of the overall conformation. It confirmed many deductions drawn from earlier work — the presence of four trx-fold domains plus a distinct **x**-region linking the **b′** and **a′** domains, and a distinct, non-trx-fold C-terminal region (Fig. 3). The most striking new finding was that the three key functional domains **a**, **b′** and **a′** are essentially co-planar and form a horseshoe with the dithiol/disulfide active sites of the **a** and **a′** domains and a hydrophobic surface from the **b′** domain lining the interior. This immediately suggested that substrates for oxidative protein folding bind within this horseshoe and the authors estimated that a folded protein or domain of approximately 100 residues could be accommodated in this space.

However, no structures of *S. cerevisiae* PDI/ligand complexes have been published and the most significant additional structural insight into the activity of this PDI comes from a subsequent lower resolution structure (PDB id: 3B0A) obtained from crystals generated at higher temperature [13]. In this structure, the most significant change from the original structure is a rotation of the **a** domain relative to the remainder of the molecule so that the two active sites no longer face each other across the tips of the horseshoe. Protease-sensitivity was used to show that this twisting at the **a**-**b** domain interface (the blue-green interface in Fig. 3) is also observed in solution, and its functional significance was demonstrated by generating mutants in which adjacent domains were cross-linked and constrained by engineered disulfide bonds. Introduction of an engineered bond into the **a**-**b** interface reduced PDI activity by 60–70% whereas similar inter-domain bonds linking **b′** and **a′** domains reduced activity by only 25%. This work [13] provides persuasive evidence that inter-domain flexibility at the **a**-**b** domain interface is functionally significant for *S. cerevisiae* PDI but, sadly, there has been very little work on this PDI in solution to develop this conclusion, in contrast to work on PDIs from other sources (see below).

Relatively recently, the first crystal structures of mammalian PDI have been published [14]. Crystals of the **abb′xa′** fragment (i.e. lacking the C-terminal **c**-region) of human PDI were generated in presence and absence of DTT and structures were solved to resolutions of < 3 Å allowing reduced (PDB id: 4EKZ) and predominantly oxidized hPDI (PDB id: 4EL1) to be compared. Analysis of these structures and their comparison with each other and with the higher resolution structure of yPDI (2B5E) provides interesting insights. The three structures are very similar and notably there is little difference between the three structures in the relative orientation of the **b**-**b′** domains, but the **a** and **a′** domains show different orientations relative to this base. The distance between the α-carbons of the exposed active site Cys residues in the **a** and **a′** domains is 26.7 Å in yPDI, 27.6 Å in reduced hPDI and 40.3 Å in oxidized hPDI. The differences between reduced and oxidized crystal forms of hPDI are primarily found in the **b′xa′** region — the **a**-**b** domains can be superimposed very easily with an rmsd (root mean square difference) value of 1.9 Å. However, in the oxidized structure, there is significant rotation of the **b′xa′** region relative to **ab** and significant rotation of **a′** around the **x**-linker, leading to the increase in distance between the active sites noted above and to greater exposure of the ligand-binding region within **b′**. Wang et al. [14] propose that this redox-dependent difference reflects a structural change linked to the functional cycle of PDI as catalyst of oxidative protein folding. It is worth noting that in the high-resolution structures of yPDI, the redox-state is mixed — the active site in the **a** domain appears to be oxidized while that in the **a′** domains is reduced. It is also worth noting that in the reduced hPDI structure (4EKZ) a molecule of DTT is present bound to the **b′** domain, so it is not clear if the differences between this and the structure observed in absence of DTT are exclusively the result of a difference in redox state.

As with all crystal-derived structures, the results raise two questions. Are these PDI structures significantly constrained by their specific crystal environments and contacts? And how representative are they of the ensemble of structures that are likely to occur in solution? In the ‘high temperature’ yPDI structure (3B0A) there is extensive contact between neighbouring molecules to form a crystallographic dimer in which the active site in **a′** and ligand binding site in **b′** are buried. There is good evidence for low-affinity dimerization of yPDI in solution, but it is not clear if this involves the interactions observed in the crystal state [13]. By contrast, in the original yPDI structure (2B5E), four molecules of yPDI form a tetramer around a crystallographic 4-fold axis, but the contact areas are small; they are unlikely to be relevant to function nor are they likely to constrain the conformation significantly. On the other hand, in this structure (2B5E), the **b** domain of one molecule is inserted into the ‘horseshoe’ of an adjacent molecule, interacting extensively with the **a** domain. Similarly, in the hPDI crystal structures, the **a** domain of one molecule is inserted into the hypothesized ‘binding cleft’ of the other, extensively in the case of oxidized hPDI (4EL1) and to a limited extent for reduced hPDI (4EKZ). These interactions are unlikely to be significant in solution since dimerization of hPDI in solution involves either mutual contact of active sites [55] or of **b′** domains [56] and a recent cross-linking study found dimer contacts inconsistent with the crystallographic data [57]. Nor can these interactions be regarded as modelling a substrate interacting with PDI, since there is no contact with the **b′** domain, the core of the PDI ligand-binding site.

The publication of a high-resolution structure of a complex between a PDI fragment and a peptide ligand [58] is a significant advance. This work involved PDI from the fungus *H. insolens* and it is considered in a later section.

## 7 Evidence that mammalian PDI is highly dynamic in solution

There is extensive biophysical evidence that mammalian PDI is highly dynamic in solution. The evidence comes from chemical cross-linking, proteolytic susceptibility and from a range of physical methods including intrinsic and extrinsic fluorescence, high-field NMR and small angle X-ray scattering (SAXS). The flexible nature of PDI is indicated in these studies i) by the presence of structural features in solution that cannot be reconciled with the structures determined by X-ray crystallography, ii) by the co-existence of alternative conformations (in absence of redox change) and iii) by the presence of alternative redox-dependent conformations.

In very early studies, cross-linking with bifunctional thiol-directed reagents showed that the active sites in **a** and **a′** domains (of bovine PDI) could be cross-linked by reagents capable of spanning 16 Å and by longer homologs [59]. This implies that, in solution, the active sites can approach much nearer than the 27–40 Å observed in the PDI crystal structures. This result has recently been supported by a very thorough study of intra-molecular cross-linking in oxidized human PDI with an amino-directed cross-linker capable of spanning 30 Å [57]; the study found numerous cross-links between residues in the **a** and **b′** domains and also links between Lys450(467) (in **a′**) and both the N-terminus and Lys86(103) (in **a** domain). All these cross-linked residues are > 50 Å apart in the hPDI crystal structures and hence the formation of these cross-links in solution implies extensive deviation from the crystal structures and compaction of the protein. The implication that the active sites in PDI **a** and **a′** domains approach much more closely than is observed in crystal structures is also supported by functional evidence. Araki and Nagata [60] studied the oxidation of hPDI by Ero1α *in vitro* and their observations are consistent with a model in which there is direct intramolecular transfer of electrons between active sites within a PDI molecule from the **a** to the **a′** domain.

**Fig. 4:**
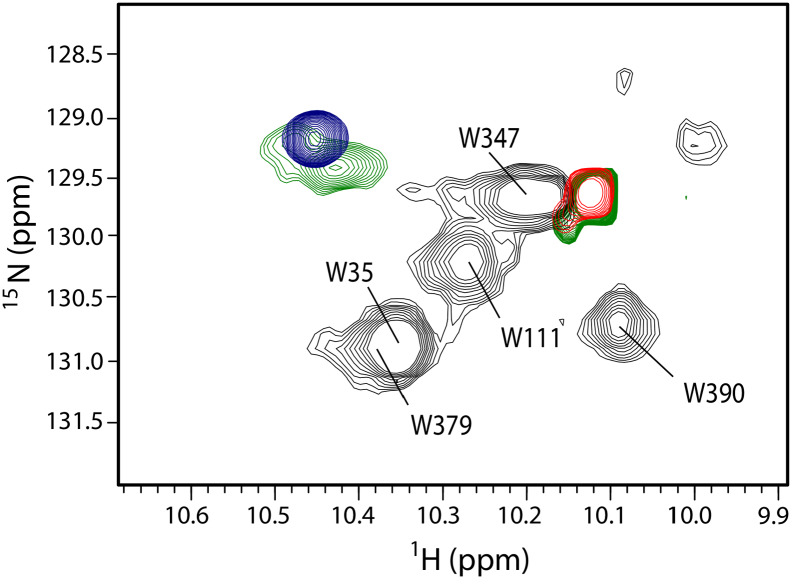
Flexibility of the **x**-region revealed by NMR spectroscopy. Overlay of the tryptophan indole regions of 600 MHz ^15^N-^1^H HSQC NMR spectra. The grey spectra are from full-length hPDI which contains 5 tryptophan residues, and assignments are shown (numbered as for the mature PDI sequence). The coloured signals are from **b′x** domain constructs which contain only one tryptophan residue, W347; the green signals are from **b′x** wild-type, the red signal from **b′x** L343A mutant and the blue signal from **b′x** I272A/D346A/D348A triple mutant. Reproduced with permission from [61].

Considerable insight into hPDI flexibility has come from exploiting the distinctive fluorescence and NMR signals from the tryptophan residue located in the **x**-linker region, W347(364). In the **b′x** fragment of hPDI, this unique tryptophan gives rise to two distinct ^15^N-^1^H HSQC NMR signals (Fig. 4), implying that the side-chain can exist in alternative environments differing in hydrophobicity [61]. However, specific mutations drive the signal to either one of the positions observed in wild-type **b′x**, either an exposed hydrophilic environment (in the L343(360) mutant) or a very hydrophobic buried environment (in the triple mutant). Intrinsic fluorescence studies and the high resolution X-ray structure of the **b′x** I272(289)A mutant (PDB id: 3BJ5) confirm these interpretations, showing that W347(364) in the **x**-linker can move freely in and out of the major binding site in the **b′** domain. Most importantly, the NMR signal from W347(364) in full-length wild-type PDI is in a position intermediate between the two environments and shows asymmetric line broadening, demonstrating that it is in conformational exchange between the two forms [61]. This conclusion was extended in studies using intrinsic fluorescence, fluorescence quenching and binding of the extrinsic fluorescent probe ANS [62] confirming that in full-length PDI, W347(364) in the **x**-linker moves between an exposed hydrophilic environment (red-shifted intrinsic fluorescence, facile quenching of intrinsic fluorescence by iodide anions, extensive fluorescence enhancement of ANS due to its binding to the ligand binding site on **b′**) and a hydrophobic environment buried within the binding site (blue-shifted intrinsic fluorescence, limited quenching by iodide and limited binding of ANS). These results imply that PDI can access in solution a structure in which the **x**-linker obstructs access to the ligand binding site on **b′**; such a structure is observed in crystals of the **b′x** fragment [61] but not in crystals of full-length hPDI [14].

**Fig. 5:**
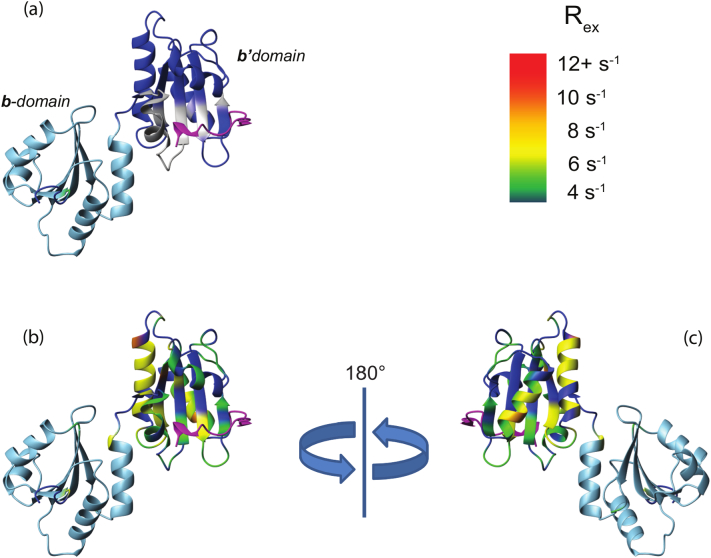
Differences in internal dynamics between the **b** and **b′** domains of human PDI. Dynamics of the PDI backbone in the **b** and **b′** domains determined from 14.1 T-derived ^15^N NMR relaxation parameters. The monomeric recombinant **bb′x** fragment of hPDI was expressed, ^15^N-labelled and purified as described [24], [56]. Spin-lattice (R_1_) and spin-spin (R_2_) relaxation rate constants were determined for backbone NH groups as described by Farrow et al. [63] and used for flexibility calculations. a) Structural model of **bb′x** based on PDB id: 2K18 and PDB id: 3BJ5, with **b** and **b′** domains shown in light and dark blue respectively, the **x**-linker in magenta and the ligand binding site determined by Byrne et al. [24] highlighted in grey. b,c) **bb′x** structure in the same orientation as in a) or rotated through 180°, coloured according to flexibility as determined by ModelFree 4.15 R_ex_ parameter [64], [65] calculated from the experimental relaxation parameters (see rainbow scale, top right). Backbone regions of low flexibility are coloured as in Fig. 5a.

NMR relaxation studies of the monomeric **bb′x** fragment of hPDI interpreted by ModelFree analysis demonstrate the dynamic nature of the **b′x** region (Fig. 5). ModelFree uses measured ^15^N NMR relaxation parameters to predict motional characteristics [64], [65]; Fig. 5b–c highlights conformational exchange contributions (high values of R_ex_) dominating throughout the **b′x** regions. Conformational flexibility in **b′** extends beyond its core ligand-binding site, encompassing much of the domain and the associated **x**-linker (Fig. 5a, cf. Fig. 5b–c), showing the ability of this whole domain to adapt to a wide variety of substrates of different sizes and shapes. Use of the **bb′x** fragment [24], [56] for this work provided the **b**-domain as an internal control and demonstrated the absence of rapid motion within this domain, highlighting the structure-function differences between **b** and **b′** domains in hPDI. However, since full-length hPDI was too large to be used for this NMR dynamics analysis, it is not known if internal mobility in **b′x** is affected by the oxidative status of **a** and **a′** domains in full-length hPDI.

**Fig. 6:**
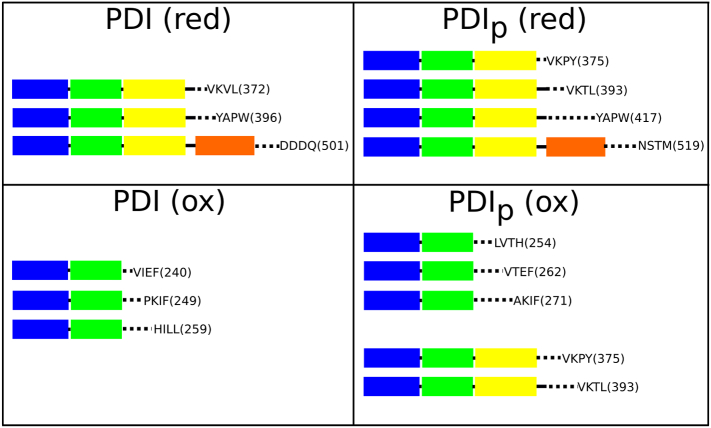
Limited proteolysis reveals differences in flexibility between reduced and oxidized hPDI. Recombinant human PDI and its close homolog, the pancreas-specific form PDIp, were subjected to limited digestion by chymotrypsin in reducing and in oxidizing conditions. The proteins (15 μM) were incubated with chymotrypsin (75 nM) at pH 7.6, 37 °C for 120 min in presence either of 10 mM dithiothreitol or 10 mM diamide. The digestion mixes were subjected to electrospray ionization mass spectrometry, analyzing products of mass > 20 kDa (see Walker [66]). All the products detected were found to contain the intact **a-b** region despite the presence of multiple potential chymotryptic cleavage sites in these domains. Products are identified in the figure by their C-terminal residue (i.e. the site at which enzymic cleavage has occurred, numbering as for the unprocessed translation product) and intact domains are coloured according to the scheme of Fig. 1. In reducing conditions all the products contain intact **abb′x** domains with cleavage occurring only at sites within **a′** and **c** domains; in oxidizing conditions the fragments are smaller with most fragments deriving from cleavage within the **b′** domain although, for PDIp, there are two products found in both reducing and oxidizing conditions.

That the **x**-linker and its junctions with adjacent **b′** and **a′** domains provides a site of extensive flexibility has also been highlighted by proteolysis studies which indicate that mammalian PDI is most susceptible to protease cleavage at and around this linker and in the neighbouring **a′** domain [18], [62]. Subsequently, specific cleavage sites have been identified (Fig. 6) showing that in reduced hPDI, chymotrypsin cleavage only occurs at the start of the **a′** domain, just before the active site in the **a′** domain and in the **c**-region [66]. By contrast, in oxidized hPDI, additional cleavage sites are observed in the **b′** domain, highlighting increased exposure and flexibility of **b′** in the oxidized state, consistent with the major change observed between the reduced and oxidized crystal structures [14]. Interestingly, this difference in proteolytic susceptibility between reduced and oxidized hPDI is not so clearly observed in the pancreas specific homolog PDIp (Fig. 6). In this homolog, some proteolytic fragments are produced specifically in oxidizing or reducing conditions, but two fragments are detected after chymotryptic digestion in both conditions. Other data from fluorescence and circular dichroism spectroscopy confirm that this homolog has a more limited redox-linked conformational change than that observed for hPDI [66].

Further exploration of the flexibility of human PDI in solution occurs in a study of novel specific PDI inhibitors [67]. In this work, a number of small molecule non-peptide ligands (bepristats) were developed that bind to the **b′** domain of hPDI and inhibit thiol:oxidoreductase activity involving a protein disulfide substrate (insulin), but enhance activity involving a small molecule disulfide (di-eosin-GSSG). The researchers inferred that there is allosteric communication between the ligand-binding site and the catalytic sites and showed, by a combination of solution techniques, including small-angle X-ray scattering, that binding of bepristats shifts the active-sites to a more reduced state, induces movement of the **x**-linker region, and leads to compaction of the PDI molecule.

## 8 Analysis of PDI from the thermophilic fungus *Humicola insolens*

No structural comparisons have been published for *S. cerevisiae* PDI in reduced and oxidized state, but valuable insight comes from extensive studies of PDI from a thermophilic fungus *Humicola insolens.* In this case, NMR data indicated that substrates bind to a hydrophobic surface spanning the **b′** and **a′** domains which becomes more solvent-exposed on oxidation, and also that the redox state of **a′** influences the dynamic properties of the **b′** domain (based on H-D exchange and NMR relaxation parameters) [68]. Furthermore, small-angle X-ray scattering analysis showed a significant redox-dependent conformational change, specifically a reorientation of **a′c** relative to **abb′** domains and a reduction in contact between **b′** and **a′** domains on oxidation [69]. The structures of the **b′xa′** fragment of this PDI were determined by X-ray crystallography in the reduced (PDB id:3WT1) and oxidized (PDB id:3WT2) states [70]. Only a limited difference in relative orientation of the domains was found (both were ‘open’), unlike the ‘open-to-closed’ transition observed in oxidized and reduced human PDI structures. However, the researchers concluded that crystal packing constraints inhibited the observation of the expected structural transition.

Recently, work on PDI from this fungus has provided the first high resolution structure of a PDI:ligand complex [58]. Interaction in solution between the PDI **b′xa′** fragment and the unfolded protein, α-synuclein, was characterised by high-field NMR, indicating that the oxidized PDI fragment interacts preferentially, and identifying a region of α-synuclein that specifically interacts with the ligand-binding site. Based on this, a peptide fragment of α-synuclein was synthesized and co-crystallized with the oxidized PDI **b′xa′** fragment (residues 208–449) and the structure solved to 1.6 Å resolution (PDB id:5CRW). Two alternative modes of binding were observed in the crystals, but a major binding site on the **b′** domain was defined and it was shown that ligand binding led to a change in relative orientation of the **b′** and **a′** domains. Yagi-Utsumi et al. [58] did not compare the site defined by their X-ray and NMR data to the major ligand binding site on the **b′** domain of human PDI determined previously [24], [61], [71]. However, it is clear that, in solution, both hPDI and *H. insolens* PDI show an extensive redox-dependent conformational change focussed around the **x**-linker and the interface between **b′** and **a′** domains, consistent with that observed in X-ray crystal structures of reduced and oxidized hPDI.

## 9 Modelling the flexibility of PDI

The construction of PDI as a linear sequence of trx-fold domains provides the potential for inter-domain flexibility and such flexibility would provide some rationale for the extraordinary versatility of PDI functions and the diversity of its interactions. This flexibility would be entirely consistent with the X-ray-derived structures, which show that the inter-domain contact areas are relatively small, especially at the **a**-**b** and **b′**-**a′** domain interfaces. However, the recognition that flexibility is highly likely does not provide any useful insight into the precise nature of PDI dynamics, and the extensive indications of PDI flexibility in solution do not provide high-resolution structural data. A rapid method for simulating realistic protein motion at residue or preferably atomic resolution is required.

Modelling of protein internal motion is conventionally performed by molecular dynamics (MD) simulations, but for a protein of the size of PDI (c. 500 residues) this is extraordinarily demanding in terms of computational resources. Furthermore, most MD simulations are limited to motion on < 1 μs timescales, whereas functional conformational changes involving relative motions of domains may occur many orders of magnitude more slowly [72]. For this reason, Jimenez-Roldan et al. [73] developed a rapid and computationally parsimonious approach to modelling protein flexibility. The approach uses rigorous criteria to represent the protein as a network of rigid clusters and flexible regions and then normal mode analysis to identify low frequency modes of motion of this network. It then applies geometric motion simulation to integrate these and explore the flexible motion of the protein along these normal modes [73]. The approach defines the scope for internal motion of a protein and has the essential attribute that it rapidly generates motion trajectories defining all atom positions in PDB format, while maintaining realistic local atomic geometry and inter-atomic constraints.

We have applied this method to explore the flexibility of yPDI [74] and hPDI [75], and summarise the results here; full descriptions of the simulated trajectories, expressed as a series of PDB data files, plus other analyses can be found on the associated web-site [75]. The analyses were based on the original high resolution yPDI structure 2B5E [12], and the oxidized (4EL1) and reduced (4EKZ) hPDI structures [14]. (Note that the X-ray derived structures of hPDI are missing short regions of sequence; 4EL1 (oxidized) is missing residues (250–254) and (320 − 323), whereas 4EKZ (reduced) is missing (240–244) and (322 − 323)). We did not model or constrain these missing regions, but the flexibility simulations did not move the ‘free’ chain ends to unfeasible distances. The results of these simulations are striking and are broadly similar for human and yeast PDI. The basic geometry of the core β-sheet within each trx-fold domain is fully maintained through the simulations so that the protein motion can be represented, to a first approximation, as relative motion of domains (the limited flexing of the core β-sheets was comparable to that found by conventional MD [74]).

**Fig. 7:**
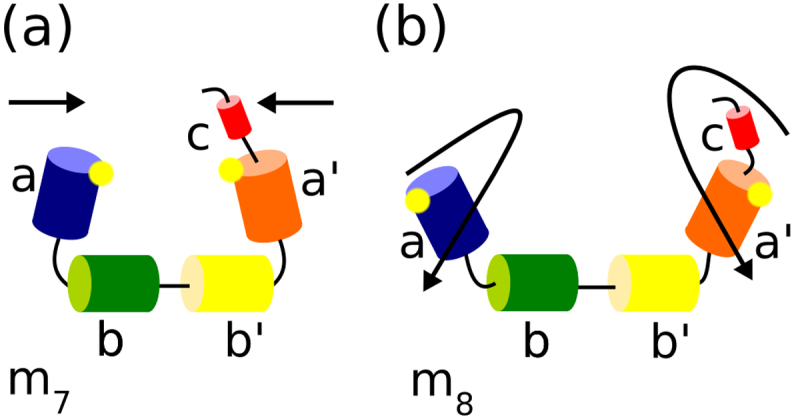
Schematic of low frequency modes of domain motion determined by flexibility analysis. PDI is shown as linked domains, represented as cylinders coloured as in Fig. 1, with active sites indicated as yellow dots. a) Mode 7, m_7_, the lowest frequency mode of flexible motion (modes 1–6 represent simple translations and rotations) is dominated by the hinge bending (tilting) of the outer domains **a** and **a′** towards and away from each other with the **bb′** region relatively fixed. b) Mode 8, m_8_, the next lowest frequency mode of flexible motion, is dominated by the rotation of domains **a** and **a′** (twisting) relative to **bb′**. Comparable cartoon representations of modes 9 to 11 for yeast PDI are shown in the supplementary material of Römer et al. [74].

In normal mode analysis of protein motion, modes 1–6 represent rigid translational motion of the molecule in three-dimensional space and rigid rotation of the molecule around three perpendicular rotational axes. So the lowest frequency (most facile) mode of internal motion is termed mode 7 (m_7_) and modes 8, 9, 10 etc. are the next lowest frequency modes of internal flexibility. Flexibility analysis has focussed on modes 7–11 (m_7_–m_11_) since these five most facile modes of internal motion encompass the great majority of the capability the protein has for internal flexible motion [73]. For all the high resolution PDI structures analysed, m_7_ involves the hinge-bending motion of the outer domains **a** and **a′** relative to the **b**-**b′** base (Fig. 7) so that the outer domains flex inwards and outwards compared to their positions in the crystal structure. The next lowest frequency mode (mode 8, m_8_) involves the rotation of **a** and **a′** relative to the **b**-**b′** base. Higher frequency modes m_9_–m_11_ combine hinge-bending and rotational motion, so that it is convenient to characterise the flexible motion in terms of changes in orientation of adjacent domains – a ‘tilt’ angle representing hinge-bending, and a ‘twist’ angle representing relative rotation.

**Fig. 8:**
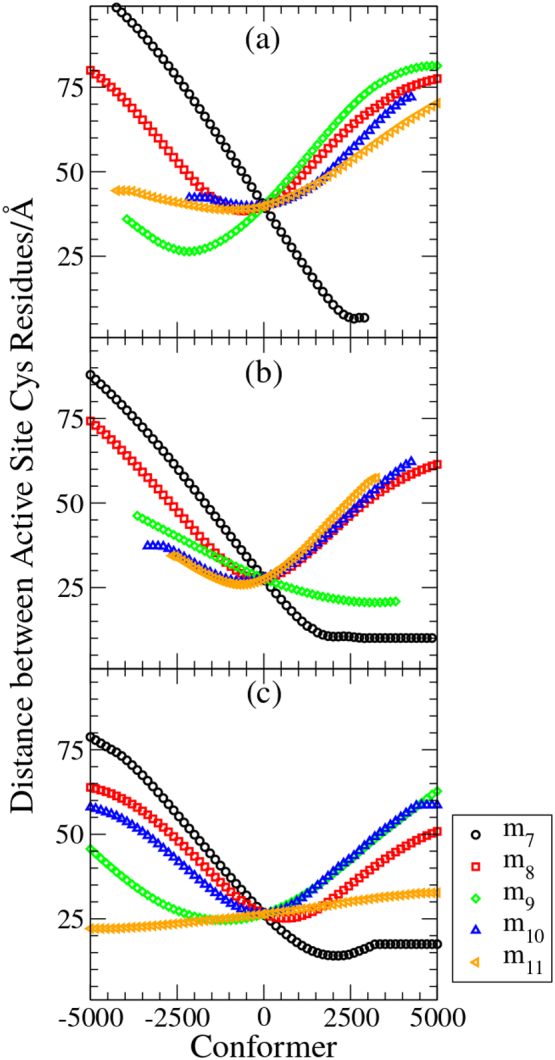
Variation in distance between active site Cys residues through flexible motion. Distances between the α-carbon atoms of the exposed active site Cys residues in the **a** and **a′** domains were determined through simulations of the lowest frequency flexible modes of motion. Conformer zero represents the initial structure observed in the crystal structure of a) oxidized human PDI (4ELI), b) reduced human PDI (4EKZ) and c) yeast PDI (2B5E). Conformers to + 5000 and − 5000 represent structures determined through the flexibility simulations in modes m_7_ to m_11_. For most of the flexible modes, the combination of tilting and twisting of domains ensures that the distance between sites can both increase or decrease (see Fig. 7). But m_7_ corresponds to domains **a** and **a′** moving towards (+ ve conformers) and away from (− ve conformers) each other, so the distance between active sites declines to low values in the + ve conformers as the domains approach; in b) and c) protein structural constraints limit the closest approach and the simulations show that a minimum distance is defined as the simulations progress to generate conformers >+2000, whereas in a) (oxidized human PDI) there are fewer structural constraints and the simulation progresses until steric contact is made between the approaching domains, at which point the simulation halts.

The hinge-bending of domains **a** and **a′** in m_7_, the most facile (lowest frequency) mode of motion, allows an opening and closing of the PDI ‘horseshoe’ such that the volume enclosed within it varies very considerably and the distance between the Cα atoms of the active-site residues in the **a** and **a′** domains ranges from close contact to a great distance (Fig. 8); for yeast PDI the range of distances is 15–80 Å (cf 27 Å in the crystal structure) whereas in reduced hPDI the Cα atoms of the active site residues can approach to within 10 Å and in oxidized hPDI the closest approach can be as low as 7 Å. The distance between Cα atoms linked by a disulfide bond is generally in the range 6 ± 1 Å [76] so this result suggests that, at closest approach, a disulfide bond could be formed between the active sites in the **a** and **a′** domains of hPDI.

**Fig. 9:**
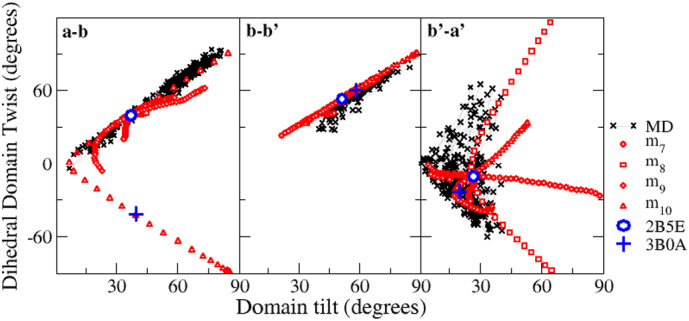
Domain orientations during flexible motion of yeast PDI analysed by ‘tilt’ and ‘twist’ angles at each domain interface. The relative orientation of neighbouring domains through simulation of flexible motion of yPDI (2B5E) is presented as a plot of twist angle v. tilt angle for each domain interface (from Römer et al. [74]; see this paper for a full definition of tilt and twist angles between neighbouring domains. Data from a 30 ns molecular dynamics simulation of motion are also shown; domain orientations in the starting structure (2B5E) are indicated by a blue circle and those in the lower resolution yPDI structure (3B0A) are indicated by a blue cross, demonstrating that the latter structure is readily generated from 2B5E by flexible motion in low frequency modes.

The net effect of the five lowest frequency modes of motion m_7_–m_11_ is that there is some inter-domain motion at each inter-domain interface but with highly different characteristics. We demonstrate this here, represented in plots of ‘tilt’ against ‘twist’ angles, using the data for yPDI [74] (Fig. 9) but similar results are observed for hPDI [75]. The simulations show that, at the **b**-**b′** interface there is limited hinge-bending motion and no rotation, comparable to that of a stiff knee-joint. At the **a**-**b** interface there is free hinge-bending plus some limited rotation, comparable to that of an elbow joint. By contrast, there is extensive bending and rotational motion at the **b′**-**a′** interface, comparable to that of a shoulder joint. Overall, the three structures (yPDI and reduced and oxidized hPDI) show similarity in potential inter-domain motion and so have similar scope to open and close and to ‘twist’ to take up conformations different from the starting crystal conformation.

**Fig. 10:**
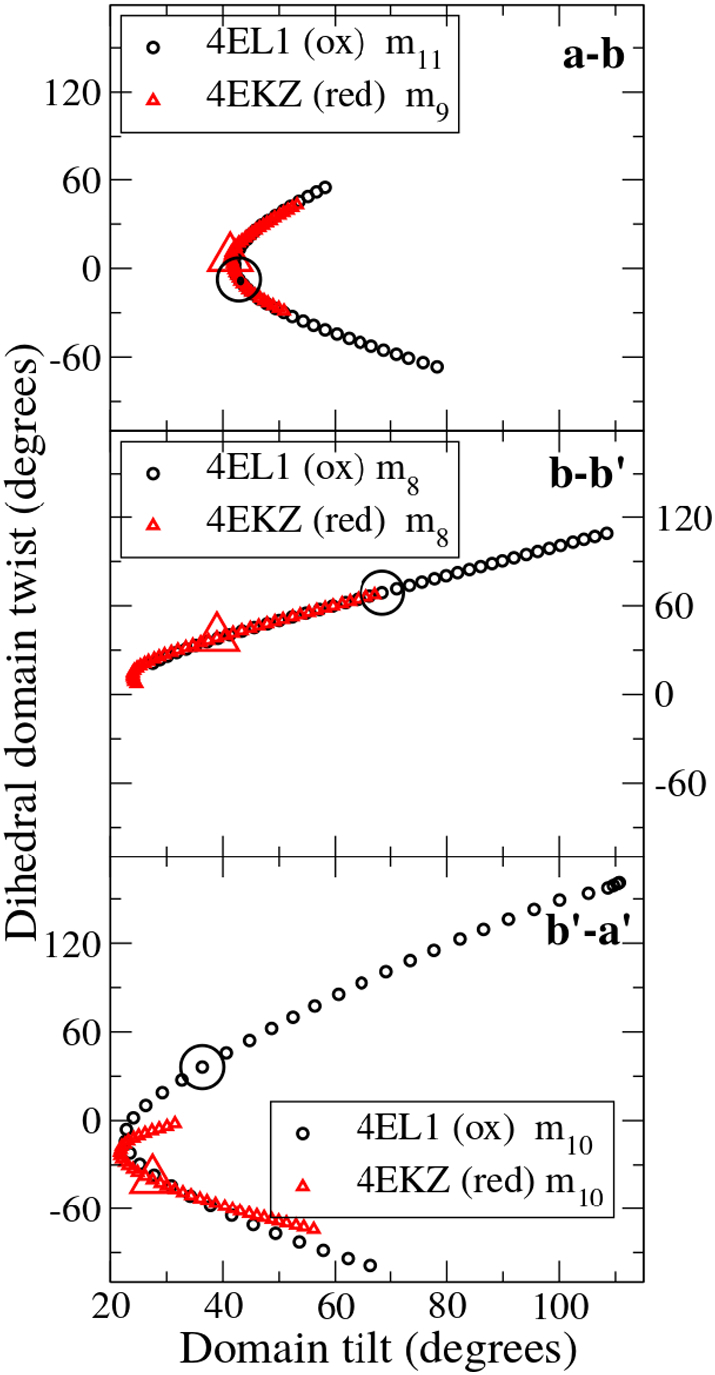
Flexible motion modelled on one hPDI crystal structure shows facile conversion to the alternative hPDI crystal structure. Flexible trajectories of specific low frequency modes interconvert the domain orientations in alternative human PDI structures. The large circles indicate the initial orientations of oxidized human PDI (4EL1) and the large triangles the initial orientations of reduced human PDI (4EKZ) at the **ab** (top), **bb′** (middle) and **b′a′** (bottom) domain interfaces. For the **ab** interface the initial conformations are very similar and the figure shows that m_11_ for one structure and m_9_ for the other trace out the same trajectory and precisely interconvert the structures. For the **bb′** interface, the initial conformations are different but flexible motion of m_8_ readily interconverts the structures. For the **b′a′** interface, the more open oxidized structure can readily reproduce the conformation found in the reduced structure by motion along m_10_ but the more constrained nature of the latter means that the converse does not hold completely.

Most strikingly, the range of flexible motion in low frequency modes, starting from either of the two hPDI structures includes the domain orientations found in the other structure (Fig. 10), so that the two crystal structures represent alternative members of an ensemble of structures which can be accessed from each other by low-frequency flexible motion. A similar result is found in the case of yeast PDI (Fig. 9) where motion at the **a**-**b** interface in m_10_, directly converts the inter-domain orientation of the starting structure (2B5E) into that observed in the alternative crystal form (3B0A). In every case, the simulation is not provided with information on alternative structures, so this ‘interconversion of structures’ by our flexibility simulations strongly validates the plausibility of the trajectories.

The simulations [74] show that there is intra-domain flexibility also, and this is most marked in the **b′** domain, suggesting that this domain, which forms the core of the PDI ligand-binding site, can flex to optimize its interactions with ligands (see Fig. 4 of [74]). This conclusion is independently supported by the NMR data shown above (Fig. 5).

To validate the simulation approach, the motions of yPDI predicted in the flexibility simulations were checked for consistency with those found in a short (30 ns) MD simulation. There is good consistency between the approaches (see e.g. Fig. 9), but the flexibility simulations go further; for example the MD simulation generated distances between the active sites in the range 22–68 Å, rather than the 15–80 Å found by flexibility modelling, and the MD simulation did not predict the combination of bending and domain rotation to generate the domain orientations found in the alternative yPDI crystal structure (3BOA). Nevertheless, the structures generated by the flexibility simulation are not implausible; when the most ‘closed’ of these (with the shortest distance between active sites in **a** and **a′** domains) was used as the initial state for an MD simulation, it was found to be relatively stable and led only to minor local changes in conformation [74]. Hence this ‘closed’ conformation represents a local conformational minimum but one not found from the starting crystal structure in a conventional short MD simulation.

It is also interesting to compare the results of simulations of human PDI flexibility with those of a recent molecular dynamics simulation based on the same hPDI structures [77]. The results of Yang et al. [77] are presented mainly in terms of distances between mid-points of domains rather than domain orientations, but the key result is that, while the distances between adjacent domain mid-points remain roughly constant, those between non-adjacent domains diminish through the 300 ns of MD simulation so that overall the simulations generate highly compact conformations. In these compact conformations, the **a** and **a′** domains are much closer than in the original structures and new interactions are formed between non-adjacent domains. Desmond et al. [75] have also analysed distances between domain mid-points through the course of flexibility simulations and, as in these MD simulations, find that the **a** and **a′** domain mid-points approach to within 30 Å. Most strikingly, the MD simulations [77] show the sulfur atoms at the active sites in **a** and **a′** domains approaching to within 5.4 Å. This distance is consistent with direct covalent bonding between the groups and Yang et al. [77] also show mutational analysis supporting the idea that direct redox interactions between the active sites is feasible. Hence the prediction by flexibility analysis of close approach of active sites in solution (Fig. 8) is also made by MD analysis [77] and is supported by cross-linking data and by functional analyses (see above) [57], [59], [60].

It should be noted that the MD study of Yang et al. [77] involved simulating 300 ns of motion from each starting structure and hence each simulation would have consumed > 36,000 CPU (central processing unit) hours compared to the 1 CPU hour involved in simulations of the lowest 5 modes of flexible motion. Nevertheless, the results are generally highly consistent and define the same major features of motion. While it would be impractical in terms of computing time to model the motion of every structure of a PDI family member by MD, the greater economy of the flexibility approach means that such modelling is feasible, and we have modelled motion of ERp57 (PDB id: 3F8U), ERp27 (PDB id: 4F9Z) [78] and ERp44 (PDB id: 2R2J) (see [75]).

In summary, modelling of PDI flexibility produced results consistent with experimental data on both fungal and mammalian PDI. Specifically, the simulations predict i) the close approach of the active sites consistent with cross-linking and functional data on mammalian PDIs, ii) the domain re-orientations that interconvert the reduced and oxidized crystal conformations of hPDI and that interconvert the high and low temperature crystal structures of yeast PDI, and iii) the extensive relative motion of the **b′** and **a′** domains, consistent with biophysical data for human and *H. insolens* PDI. But the modelling is not only consistent with experimental data; it goes beyond it by providing plausible trajectories for motion of the protein, fully defined at the atomic level in terms of PDB co-ordinates. We conclude that the method provides a valuable basis for exploring the full scope for motion of PDI and similar large multi-domain proteins and for making predictions that can be tested experimentally.

The full dataset from simulations of flexible motion of human and yeast PDI, including intermediate structure files, is available at Desmond et al. [75], allowing other researchers to interrogate the simulations and to test their predictions against a wide range of experimental data.

## 10 What is the functional significance of PDI flexibility?

The most striking aspect of the flexibility of PDI revealed by a range of solution data, by the few X-ray structures and by the flexibility simulations, is that there is extraordinary potential for change in the relative orientation of the **a** and **a′** domains relative to **b′b′**. Whereas the MD simulation of human PDI motion highlighted a transition to a more compact structure in every case, flexibility simulations show the potential for both ‘opening’ and ‘closing’ motions. This flexibility permits a vast change in the volume included between the domains, in the distances between key sites, (specifically the redox-active sites in **a** and **a′** domains and the core ligand binding site in **b′**) and in the relative orientation of these sites. Flexibility of this kind provides a rationale for the extreme diversity of PDI interactions with folding substrates and protein partners. Whereas PDI alone in solution may be more compact than observed in the crystal structures, it is very probable that its complexes with large protein ligands cover a wide range of structures, both more ‘open’ and more ‘closed’ than observed in crystals.

Furthermore, the flexibility simulations and the NMR data (Fig. 5) indicate extensive intra-domain flexibility in the **b′** domain, implying that the promiscuous ligand-binding site in this domain can flex to optimize its interactions with ligands. Since all information on PDI/ligand interactions highlights the same site on the **b′** domain as the core of the ligand-binding site, it appears that PDI redox function in transferring electrons from a reduced protein substrate to an oxidant partner such as Ero1 or Gpx7 requires dissociation of the first ligand before the second can bind. Facile flexible motion of PDI is likely to enable this ligand exchange. Differences in conformation between oxidized and reduced states of PDI imply that flexible motion between such conformations could be a key aspect of the PDI functional cycle in oxidative protein folding.

However, in the absence of high-resolution structural and dynamic data on PDI/ligand complexes, such conclusions remain general rather than specific. While it is tempting to conclude that overall conformational change in PDI is driven by changes in redox state, leading to changes in ligand-binding properties, it is equally logical to suggest that changes are primarily driven by ligand-binding. In fact, it appears that redox state and ligand-binding are linked functions in PDI, but as yet no detailed thermodynamic analysis of this linkage has been undertaken. In its absence, it is risky to provide models linking PDI conformational change causally to functional transitions. PDI is clearly a complex machine, but the increasing weight of evidence shows that it is not a hard machine that switches sharply between precisely-defined structures. Rather it is a soft machine that can access a wide ensemble of states from which particular sub-ensembles are selected on the basis of various factors including redox state and ligand-binding and dissociation.

This review has surveyed the experimental data that indicate flexibility in PDI and has shown that rapid simulation of PDI flexibility provides models of motion consistent with these data but with far greater structural detail. But at the same time, the review highlights the paucity of high-resolution structural data that could provide a real model of how flexibility underpins PDI's functional interactions with its ligands and partner proteins. Further experimental data are urgently required.

## 11 Forward look: experimental approaches for defining better the flexibility of PDI in solution

Currently models for PDI function are being built based on a very small number of high resolution X-ray derived structures. A greater number and variety of high-resolution X-ray structures and (especially) of PDI/ligand complexes would provide hugely valuable additional insights (though possibly at the cost of complicating the picture). In particular it would be helpful to have structures of PDI in complex with a long-term protein binding partner, either with a ‘stalled’ incompletely folded protein substrate or in one of the functional complexes (prolyl-4-hydroxylase and microsomal triglyceride transfer protein) in which PDI acts as a long-term chaperone.

But other methods need to be pushed forward in order to provide dynamic high-resolution data for the protein in solution. A range of NMR approaches are available [79], [80] and in principle these approaches can provide information on all NMR-visible backbone and side-chain atoms, but these methods are challenging for a protein of the size of PDI (c. 500 residues) comprising 4 relatively flexible domains.

Cross-linking combined with mass spectrometric detection of cross-linked peptides has demonstrated its potential [57]; to be most effective, it should be applied with a range of homologous cross-linkers differing in span length and in conditions where the presence of protein dimers is rigorously excluded, so that all observed cross-links are intramolecular. This method has the benefit that it can provide information defining the closest approach distance for multiple sites simultaneously (depending on the chemistry of the cross-linker used) but it lacks a time dimension, identifying sites that approach each other at any time within the reaction time-window.

Alternatively, Förster resonance energy transfer (FRET) methods could be applied. The technology for single-molecule FRET is now well-developed and time-resolved approaches are available that can monitor over time the change in distance between labelled sites [81], [82]. This method is quite demanding – it requires the careful selection of labelling sites and controls to ensure that the introduced labels do not perturb the system under study. At present there are few useful donor/acceptor pairs with characteristic Förster distances in the 10-50 Å range that is required for determining intramolecular flexing in a protein of the size of PDI. Nevertheless, by selecting a small number of labelling sites at strategic positions around the protein, and combining the information from different pairs of sites it should be possible to triangulate and to build up a time-resolved picture of the dynamics of the whole protein.

Whether these or other experimental methods come to the fore, it will be essential to use rapid simulation of flexibility in order to have productive interplay between modelling and experiment. The challenge of understanding the functional significance of flexibility in the multiple roles of PDI is such that all available tools will need to be applied.

## Transparency document

Transparency document. Image 1
